# Management of masseter spasm after induction of general anaesthesia in a patient with depressive disorder on selective serotonin reuptake inhibitor treatment

**DOI:** 10.12669/pjms.40.11.9044

**Published:** 2024-12

**Authors:** Samina Ismail, Syeda Rimza Ali

**Affiliations:** 1 Samina Ismail, FCPS, Department of Anaesthesiology, Aga Khan University Hospital, Karachi, Pakistan; 2 Syeda Rimza Ali, MBBS, Department of Anaesthesiology, Aga Khan University Hospital, Karachi, Pakistan

**Keywords:** Masseter spasm, general anaesthesia, muscle relaxants, depression, Sertraline

## Abstract

Masseter spasm after induction of anaesthesia can be generally defined as a marked difficulty in manual mouth opening that interferes and impedes direct laryngoscopy and tracheal intubation without the presence of temporomandibular joint dysfunction. Several factors have been implicated in the literatures responsible for causing masseter spasm including use of non-depolarizing muscle relaxants, selective serotonin reuptake inhibitor and anxiety. Our case is the first to report masseter spasms with the use of Cis-atracurium in a patient on antidepressant depressant treatment with sertraline and having extreme preoperative anxiety. Anaesthesiologist should be aware of this complication when dealing with anxious patients on antidepressant therapy during induction of anaesthesia. Masseter spasm with locked jaw, can be a potential life-threatening situation particularly in scenarios of “cannot- ventilate-cannot intubate”.

Masseter spasm after induction of anaesthesia is generally defined as a marked difficulty in manual mouth opening that interferes with and impedes direct laryngoscopy and tracheal intubation without the presence of temporomandibular joint dysfunction.^1^ This condition is critical for the anaesthesiologist to consider, as in the event of a masseter spasm, not only do direct laryngoscopy and oral intubation become difficult, but most other airway devices also cannot be placed. In such scenario, if mask ventilation becomes insufficient or difficult; this can lead to the possibility of a life-threatening situation of “cannot -intubate -cannot -ventilate.

We, hereby, present a case of bilateral masseter spasm after induction of general anaesthesia in a 66 year old female patient with endometrial carcinoma scheduled for total abdominal hysterectomy and pelvic lymph node dissection. Her previous anaesthesia history included laparoscopic cholecystectomy 15 years ago under general anaesthesia with no complications reported to the patient or family. We did not have any anaesthesia record of denoting the drug used for this anaesthesia. Her second anaesthetic exposure was for a diagnostic hysteroscopy one month ago, under general anaesthesia using nalbuphine and propofol for induction, with a laryngeal mask used to secure the airway. During this procedure, difficulty was encountered in placing the laryngeal mask, leading to multiple failed attempts, according to the anaesthesia record. However, the reason for this difficulty was not mentioned.

Patient’s current medical history included depression treated by Sertraline (total dose 150mg per day) for the last two years. The last dose of Sertraline was taken the night before surgery. The rest of the preoperative anaesthesia assessment was unremarkable with all laboratory investigations within normal range. On airway examination. patient had a normal mouth opening with more than 4cm between the upper and lower incisors. The patient did not receive any premedication on the day of surgery and appeared nervous and anxious at the time of induction of anaesthesia.

General anaesthesia was induced with Nalbuphine 8mg, Propofol 150mg and Cis-atracurium 14mg. The anaesthesiologist encountered a problem in bag and mask ventilation and attempted inserting oropharyngeal airway when it was observed that patient’s mouth opening was restricted due to bilateral masseter muscle spasm. At this point, the airway management was taken over by senior anaesthesiologist enabling successful mask ventilation, with the patient’s oxygen saturation maintained at 100%. Inhalation agents were stopped and additional propofol was administered. After three minutes of bag mask ventilation, intubation was attempted with video laryngoscope and the anesthesiologist was able to insert the blade of video laryngoscope through minimal mouth opening at the right corner of the mouth and manipulated it under vision of video laryngoscope screen.

External laryngeal pressure was applied, allowing visualisation of the arytenoids. A bougie was inserted, and the laryngoscope blade was removed to create space for a smaller endotracheal tube, which was railroaded over the bougie into the trachea. Confirmation of successful endotracheal intubation was done with chest movement, capnograph and bilateral chest auscultation for breath sounds. It was decided to re-start the use of isoflurane as during the entire time, there were no signs of tachycardia, rise of end expiratory carbon dioxide levels, hyperthermia or any other signs that indicated the presence of malignant hyperthermia. Surgery was proceeded, however masseter spasm persisted during the intraoperative period. Surgery was completed uneventfully muscle relaxants were tapered off towards the end and neostigmine with glycopyrrolate was given after checking train of four muscle twitches with nerve stimulator.

A significant improvement in mouth opening was observed with reversal of neuromuscular block, and patient was extubated fully awake. The postoperative course remained uneventful, patient had no recall of the event and did not complain of any jaw pain. In our case we did not encounter a complete locked jaw as we were able to insert the laryngoscope blade. However, mouth opening was limited to a slit and only allowed the insertion of blade with no further space for any manipulation. Without video laryngoscope screen, it would have not been possible to visualize the arytenoids to guide bougie followed by endotracheal tube through the glottic opening. The patient’s attendants were informed of the event and were provided a detailed letter documenting the masseter spasm and difficulty in laryngoscopy for future reference and one copy of the letter was kept in the file for record purposes. Signed informed consent was obtained for publishing the case for anesthesiologist awareness.

Several factors have been implicated in the literature as causes of masseter spasm. Use of non-depolarizing muscle relaxants has been implicated as a possible cause of masseter spasm.^1^-^3^ This can be a likely cause in our patient as masseter spasm completely resolved following recovery and reversal of the blockade. Atracurium,^4^ Rocuronium,^5^ pancuronium^2^ have all been shown as possible agents causing masseter spasm under anesthesia, and in all these reported cases complete recovery of spasm was observed after reversal of neuromuscular block.

Muscle rigidity can be part of the serotonin syndrome caused by excessive amounts of serotonin in the central nervous system due to SSRI (selective serotonin reuptake inhibitor) overdose^6^ Our patient was receiving long-term treatment with sertraline for depressive disorder. Previous cases have also reported Sertraline as a possible causative agent for muscle spasm in patients receiving general anaesthesia.^4^,^6^,^7^

The increased use of antidepressants among older populations is noteworthy, as they are increasingly prescribed for various indications, including osteoarthritis, fibromyalgia, diabetic neuropathy, and chemotherapy-induced peripheral neuropathy.^8^ Therefore, prevention of serotonin syndrome begins with improving education and awareness as healthcare providers need to be prepared to see more cases of serotonin syndrome and its deleterious effects.

Anxiety has also been reported as a potential cause of masseter spasm.^4^ Habitual jaw clenching in anxious patients can lead to repetitive forceful muscle contractions, which, combined with preoperative anxiety, may result in involuntary jaw clenching and exercise-induced muscle spasm.^4^ Our patient was also very anxious and did not receive any premedication.

## CONCLUSION

In summary, this case is the first to report masseter spasm associated with the use of Cis atracurium. Antidepressant depressant treatment with sertraline and preoperative anxiety can be contributing factors to this grave complication. Anaesthesiologist should be vigilant during induction of general anaesthesia in anxious patients on antidepressant therapy as masseter spasm with locked jaw, can be a potential life-threatening situation particularly in scenarios of “cannot- ventilate-cannot intubate”.
